# Preoperative neutrophil-to-lymphocyte ratio and tumor-related factors to predict lymph node metastasis in patients with pancreatic ductal adenocarcinoma (PDAC)

**DOI:** 10.18632/oncotarget.11031

**Published:** 2016-08-02

**Authors:** Lianyuan Tao, Lingfu Zhang, Ying Peng, Ming Tao, Gang Li, Dianrong Xiu, Chunhui Yuan, Chaolai Ma, Bin Jiang

**Affiliations:** ^1^ Department of General Surgery, Peking University Third Hospital, Beijing, China

**Keywords:** pancreatic ductal adenocarcinoma, lymph node metastasis, prognosis, neutrophil-to-lymphocyte ratio

## Abstract

As a poor prognosis indicator in patients with pancreatic ductal adenocarcinoma (PDCA), lymph node (LN) metastasis is of great importance in treatment. Present study was performed to evaluate the predictive value of preoperative neutrophil-to-lymphocyte ratio (NLR), Platelet-to-lymphocyte ratio (PLR) and possible clinical parameters on the LN metastasis in PDCA patients. A total of 159 operable patients with PDCA were enrolled in our study. The clinical utility of NLR and other clinical parameters was evaluated by receiver operating characteristic (ROC) curves. Overall survival analysis indicated that LN metastasis is an independent prognostic factor. The logistic analysis was used to determine the independent parameters associated with LN metastasis. Ideal cutoff values for predicting LN metastasis are 2.12 for NLR and 130.96 for PLR according to the ROC curve. Multivariate analyses indicate that NLR (HR 2.588; 95% CI 1.246-5.376; *P* = 0.011), CA125 (HR 6.348; 95% CI 2.056-19.594; *P* = 0.001) and CA19-9 (HR 2.738; 95% CI 1.151-6.515; *P* = 0.023) are associated significantly with LN metastasis independently. Preoperative NLR, CA125 and CA19-9 are useful biomarkers for the prediction of LN metastasis in PDCA patients.

## INTRODUCTION

Pancreatic cancer (PC) is one of the most aggressive malignancies, and represents a leading cause of cancer-related mortality [1, 2]. Pancreatic ductal adenocarcinoma (PDCA) was the commonest histological type of PC. Most of PC patients were diagnosed at an advanced stage and its 5-year survival rate is only 6% [1-3]. Lymph node metastasis is an independent prognostic factor associated with PC, and it is of great significance for making reasonable therapeutic strategies and selection of suitable treatment options for individual patients [4-6].

Although controversies remains regarding neoadjuvant approaches in the treatment of PC recently, it has several potential advantages over adjuvant therapy, such as earlier delivery of systemic treatment, *in vivo* assessment of response, increased resectability rate in borderline resectable patients and increased margin-negative resection rate [7-9]. It is reported that patients with potentially resectable PDAC selected to undergo neoadjuvant therapy had improved survival and longer time to recurrence, especially for those with LN metastasis [8]. Therefore, neoadjuvant is necessary for some PDCA patients, and an accurate preoperative prediction of LN status is of critical significance for the selection of treatment for PDCA.

Imaging techniques, such as endoscopic ultrasonography (EUS), computed tomography (CT), and magnetic resonance imaging (MRI), are widely used in the evaluation of nodal status in PDCA patients, however, their application are limited because of their inconsistent sensitivities and specificities findings [1, 10-15]. Some novel serum markers, such as MMP7, MUC1 and MUC2, have been proposed to detect LN metastases in PDCA patients [16, 17]. However, their clinical applications are hard to achieve for their high cost and technological problems.

Since 2005, many studies have already been aware of the predictive value of the systemic inflammatory response in the outcome of patients undergoing resection for pancreatic cancer [18-21]. Neutrophil, one of the most important part of WBCs in the systemic inflammatory response, have been recognized as key participator for metastasis based on increasing evidence [22-25]. Neutrophil-to-lymphocyte ratio (NLR), one of the most used clinical parameters for the evolution of neutrophils, was considered as a convenient marker for the predictor of poor prognosis for pancreatic cancer [26-28]. Whether NLR can predict the Lymph node (LN) metastasis of PDCA is still unknown. Therefore, we performed a retrospective analysis of predictor value of NLR and possible clinical parameters on the LN metastasis of PDCA before operation.

## RESULTS

### Patient characteristics

One hundred and fifty-nine patients who had undergone a primary attempt of a curative resection for PC were enrolled, including 100 males and 59 females ranging in age from 23 to 86 years, with a mean of 63.4 years. All patients’ diagnoses were ultimately confirmed both clinically and pathologically and LN metastases were also confirmed pathologically. General clinical factors are summarized in Table 1 and Table 3 and quantitative clinical factors are shown in Table 1. Among the 159 patients, 89 (56.0%) patients were discovered developing LN metastases during operation.

**Table 1:** Univariate and multivariate analysis of clinicopathologic variables in relation to overall survival after curative operation

Parameter			Univariate analysis		Multivariate analysis	
		Number	Hazard ratio(95% CI)	*P* value	Hazard ratio(95% CI)	*P* value
Gender	male	100				
	female	59	1.307(0.887-1.926)	0.176		
Age	<65	86				
	≥65	73	1.730(1.167-2.567)	0.006	1.493(1.045-2.132)	0.028
Albumin(g/dL)	<3.5	18				
	≥3.5	141	1.430(0.754-2.711)	0.274		
CEA(ng/mL)	<5	117				
	≥5	42	2.696(1.608-4.518)	<0.0001	1.725(1.155-2.575)	0.008
CA125(U/mL)	<35	128				
	≥35	31	1.694(0.973-2.947)	0.062		
CA19-9(U/mL)	<39	34				
	≥39	125	1.360(0.811-2.281)	0.244		
PLR	<130.96	62				
	≥130.96	97	1.217(0.720-2.057)	0.463		
NLR	<2.12	53				
	≥2.12	106	1.226(0.745-2.019)	0.422		
ABO blood type	A	55				
	AB	20	1.499(0.779-2.884)	0.226		
	B	50	0.633(0.382-1.049)	0.076		
	O	34	0.992(0.597-1.647)	0.975		
Location	uncinate process	52				
	head	71	1.727(1.078-2.768)	0.023		
	body and tail	36	1.358(0.808-2.281)	0.248		
Differentiation	poor	20				
	poor to moderate	56	0.858(0.440-1.672)	0.652		
	moderate	69	0.586(0.300-1.146)	0.118		
	well to moderate	12	0.598(0.229-1.561)	0.294		
	well	2	1.215(0.240-6.165)	0.814		
Diameter(cm)	<3.5	69				
	≥3.5	90	1.757(1.143-2.700)	0.01		
T	1	12				
	2	43	1.988(0.782-5.055)	0.149	2.133(0.888-5.122)	0.09
	3	103	2.281(0.904-5.753)	0.081	3.263(1.414-7.528)	0.006
	4	4	4.906(1.159-20.767)	0.031	7.314(2.008-26.644)	0.003
Lymph node status	Negative	70				
	Positive	89	1.586(1.035-2.431)	0.034	1.751(1.208-2.538)	0.003

NLR, Neutrophil-to-lymphocyte ratio; PLR, platelet-to-lymphocyte ratio

**Table 3:** Univariate analysis of clinical characteristics according to nodal involvement

		Lymph node-negative		Lymph node-Positive		Univariate analysis	
Characteristics		(*N*=70)	(%)	(N=89)	(%)	X^2^	*P*
Gender	male	47	67.1	53	59.6	0.97	0.325
	female	23	32.9	36	40.4		
Age	<65	36	51.4	50	56.2	0.36	0.551
	≥65	34	48.6	39	43.8		
Albumin(g/dL)	<3.5	6	8.6	12	13.5	0.94	0.332
	≥3.5	64	91.4	77	86.5		
CEA(ng/mL)	<5	60	85.7	57	64.0	9.47	0.002
	≥5	10	14.3	32	36.0		
CA125(U/mL)	<35	66	94.3	62	69.7	15.14	<0.001
	≥35	4	5.7	27	30.3		
CA19-9(U/mL)	<39	22	31.4	12	13.5	7.51	0.006
	≥39	48	68.6	77	86.5		
PLR	<130.96	38	54.3	24	27.0	12.29	<0.001
	≥130.96	32	45.7	65	73.0		
NLR	<2.12	33	47.1	20	22.5	10.73	0.001
	≥2.12	37	52.9	69	77.5		
ABO	A	26	37.1	29	32.6	0.91	0.823
	AB	8	11.1	12	13.5		
	B	23	32.9	27	30.3		
	O	13	18.6	21	23.6		
Location	uncinate process	24	34.3	28	31.5	2.25	0.325
	head	27	38.6	44	49.4		
	body and tail	19	27.1	17	19.1		
Differentiation	poor	11	15.7	9	10.1	4.23	0.376
	poor to moderate	23	32.9	33	37.1		
	moderate	28	40.0	41	46.1		
	well to moderate	6	8.6	6	6.7		
	well	2	2.9	0	0.0		
Diameter(cm)	3.5	34	48.6	35	39.3	1.36	0.243
	≥3.5	36	51.4	54	60.7		
T	1	7	10.0	5	5.6	1.18	0.758
	2	18	25.7	25	28.1		
	3	46	61.4	57	64.0		
	4	2	2.9	2	2.2		

### Comparison of the clinical variables in relationship to OS after curative operation

**Figure 1:**
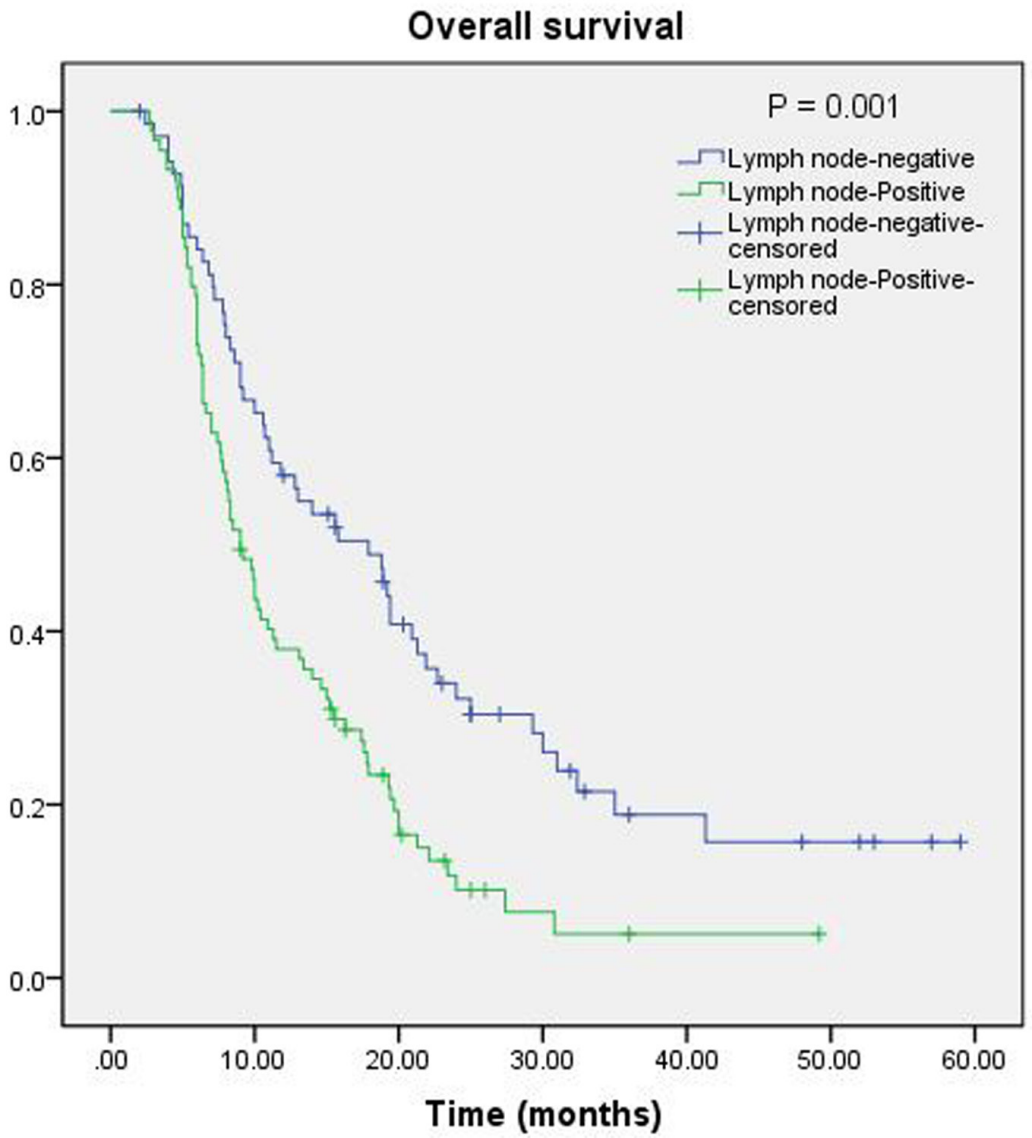
Kaplan-Meier curve for overall survival of patients with PDCA by lymph node-positive *vs*. lymph node-negative; lymph node-positive is associated with poor survival (*P* = 0.001)

In the univariate analysis, greater age (*P* = 0.006), CEA (*P* < 0.0001), tumor diameter (*P* = 0.01), T stages (*P* < 0.05), and lymph node-positive (*P* = 0.034) were significant prognostic factors for OS (Table 1). The NLR and PLR were not significant predictors of OS (*P* > 0.05 each; Table1). Moreover, in the multivariate analysis, lymph node-positive (*P* = 0.034), together with greater age (*P* = 0.003), CEA (*P* = 0.008), and T stages (*P* < 0.05), were also a significant predictor of metastasis (Table1). The association between lymph node metastasis and overall survival after surgery was also showed by Kaplan-Meier curve (Figure1).

### Preoperative NLR, PLR and clinical parameters between PDCAs with and without LN metastasis

**Figure 2:**
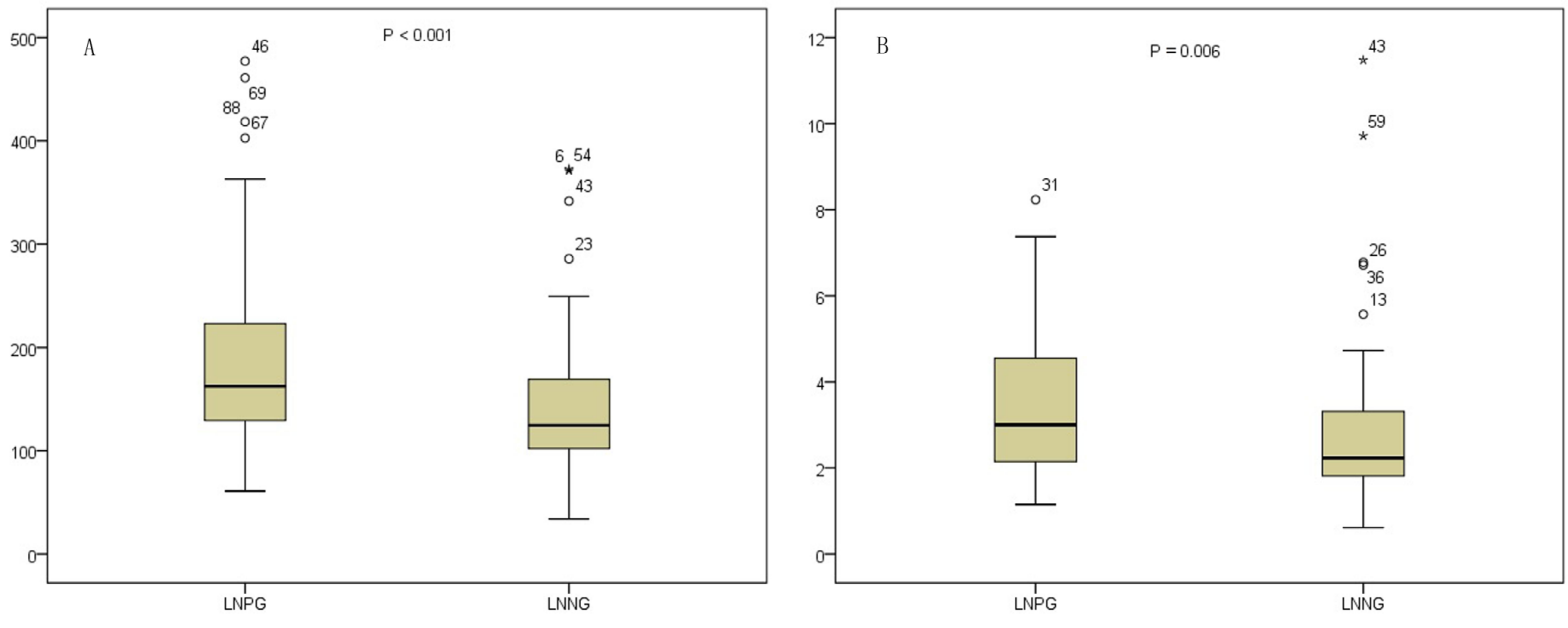
Distributions of PLR A. and NLR B. between LNNG and LNPG (LNNG, lymph node-negative group; LNPG, lymph node-positive group; NLR, neutrophil-to-lymphocyte ratio; PLR, platelet-to-lymphocyte ratio)

**Figure 3:**
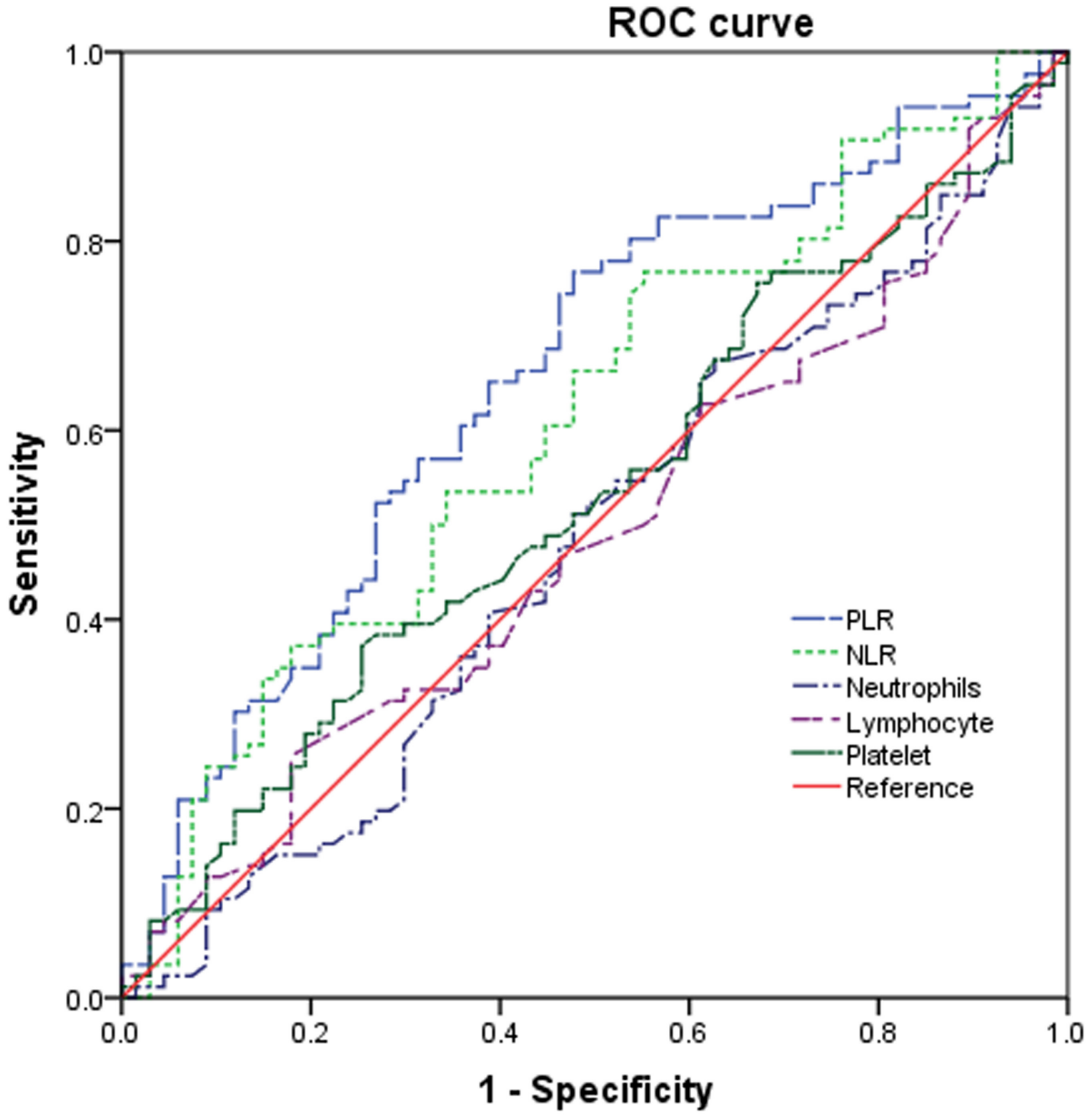
ROC curves for systemic inflammatory response markers in patients with PDCA according to lymph node metastasis (NLR, neutrophil-tolymphocyte ratio; PLR, platelet-to-lymphocyte ratio; ROC, receiver operating characteristic)

As showed in Table 2, platelet count and neutrophil count were not significantly different between those PDCA patients with nodal involvement and those without nodal involvement. However, both PLR and NLR were significantly higher in those patients with nodal involvement (*P* < 0.001 and *P* = 0.006) (Table 2 and Figure 2). The ROC curves were further used to evaluate those variables. Figure 3 shows that the AUC of PLR (0.656, 95% CI 0.568-0.743) and NLR (0.611, 95% CI 0.521-0.701) were wider than neutrophils (0.480, 95% CI 0.387-0.573), platelet (0.532, 95% CI 0.440-0.624), and lymphocyte (0.488, 95% CI 0.396-0.580), which indicated that the ability of preoperative PLR and NLR values to differentiate LN metastasis is more powerful than individual indicators of lymphocyte, neutrophils, and platelet.

**Table 2:** Comparison of quantitative clinical factors between lymph node negative group and lymph node positive group

	Lymph node-negative		Lymph node-positive		
Factors	Mean	Standard deviation	Mean	Standard deviation	*P* value
**Age(years)**	62.045	10.94	64.557	9.417	0.125
**Albumin(g/dL)**	40.9	5.796	36.313	13.167	0.208
**CEA(ng/mL)**	6.031	8.947	4.103	4.055	0.602
**CA125(U/mL)**	28.841	29.303	17.528	20.663	<0.001
**CA19-9(U/mL)**	558.313	891.257	212.849	253.049	<0.001
**Platelet (×10^3^/mL)**	210.214	70.329	203.914	63.546	0.583
**Lymphocyte (×10^3^/mL)**	1.44	0.599	1.43	0.481	0.805
**Neutrophil (×10^3^/mL)**	3.75	1.266	3.98	1.859	0.675
**PLR**	187.001	89.158	141.856	68.02	<0.001
**NLR**	3.43	1.704	2.811	1.828	0.006
**Diameter(cm)**	4.101	2.285	3.645	1.678	0.158

NLR: neutrophil/lymphocyte rates; PLR: Platelet/lymphocyte rates

According to the ROC curve plotted above (Figure 3), the cutoff values of the PLR and NLR for LN metastasis were set to 130.96 and 2.12, respectively. On the basis of the cutoff value, the diagnostic sensitivity and specificity were 74.4 and46.3%, respectively, for PLR and 76.79 and 55.2%, respectively, for NLR. Thus, we dichotomized the patients into groups of ‘high PLR (≥130.96)’ and ‘low PLR ( < 130.96)’ or ‘high NLR (≥2.12)’ and ‘low NLR ( < 2.12)’. Of 159 patients, the number of patients with high PLR and high NLR was 97 (61.0%) and 106 (66.7%), respectively. As showed in Table 1, the serum levels of CA19-9 and CA125 were statistically higher in the LN positive group (both *P* < 0.001) groups. With respect to the other quantitative clinical parameters including age, albumin, CEA, and tumor diameter, no significant difference was observed between the two groups.

### Univariate and multivariate analysis of LN metastases related factors

Univariate analysis indicated that CEA (χ2 = 9.47, *P* = 0.002), CA125 (χ2 = 15.14, *P* < 0.001), CA19-9 (χ2 = 7.51, *P* = 0.006), NLR (χ2 = 10.73, P = 0.001), and PLR (χ2 = 12.29, *P* < 0.001) showed significant differences according to the nodal involvement (Table 3). There was no significant relationship between the LN-negative and LN positive groups with respect to gender, age, albumin, ABO blood type, tumor location, tumor differentiation, tumor diameter and T staging. Therefore, five variables examined in univariate analysis (*P* < 0.05) were selected as potential independent risk factors in multivariate analysis and the results showed (Table 4) that three of these differed significantly (*P* < 0.05). Finally, we identified that NLR (HR 2.588; 95% CI 1.246-5.376; *P* = 0.011), CA125 (HR 6.348; 95% CI 2.056-19.594; *P* = 0.001) and CA19-9 (HR 2.738; 95% CI 1.151-6.515; *P* = 0.023) were independent predictive indicators of LN metastasis.

**Table 4:** Results of the clinicopathological parameters for PDCA with nodal involvement by multivariate logistic analyses

Parameters		Hazard ratio	95% CI	*P* value
**CEA(ng/mL)**	**<5**	1		
	**≥5**	1.714	0.682-4.303	0.252
**CA125(U/mL)**	**<35**	1		
	**≥35**	6.348	2.056-19.594	0.001
**CA19-9(U/mL)**	**<39**	1		
	**≥39**	2.738	1.151-6.515	0.023
**PLR**	**<130.96**	1		
	**≥130.96**	1.852	0.832 −4.123	0.131
**NLR**	**<2.12**	1		
	**≥2.12**	2.588	1.246-5.376	0.011

## DISCUSSION

Our results show that 56.0% (89/159) PDCA patients had LN metastasis when diagnosed. Our analysis show indicated LN metastasis is a independently prognostic factor for overalls survival, which is agree with many previous studies and summed in a meta-analysis [6]. As a poor prognosis indicator, it is also a key preoperative factor for the selection of neoadjuvant therapy for PDCA patients [8]. Therefore, it is of great importance to find preoperative predictive indicators of LN metastasis in patients with PDCA.

Our data show that NLR and LN metastasis are closely related (*P* = 0.011). Inflammation has been reported to increase the risk of serious cancers, such as colorectal cancer, skin cancer, and lung cancer [29-33]. As the major part of WBCs, neutrophils play an important role in tumorigenesis and progression through secreting many types of cytokines, including IL-1, IL-8, granulocyte-macrophage colony-stimulating factor (GM-CSF), and TNF-α [29]. It has been pointed out that neutrophils have a crucial role in tumor metastatic progression [22-24]. Zhang's study indicates that the abundance of circulating tumor-associated neutrophils in advanced cancer patients contributes to the circulating tumor cell survival and metastasis by suppressing peripheral leukocyte activation [25].

It is has been reported that through interaction with neutrophils, tumor cells could be brought to the endothelium, which is an essential step in LN metastases. Wculek and Malanchi clarify the role of mature neutrophils as mediators of metastatic initiation in breast cancer models [24]. Studies have indicated that tumor cells could activate and adhere to neutrophils directly through integrin [34, 35]. Integrin αV/β3, which is expressed by CTCs, could bind to surface receptors of neutrophils [36-39]. In addition, neutrophils could enhance indirect binding through paracrine of interleukin-8 and matrix metalloproteinase [40, 41]. Neutrophils also play a key role in adhesion of tumor cells to the lymphatic endothelium, which would bind to an endothelial cell if the endothelial is also sufficiently activated [42, 43]. Therefore, neutrophils might serve as mediators of the LN metastasis.

CA19-9 and CA125 are common serum markers for tumor, both are widely used in the clinic. CA19-9 is the most used serum marker in the diagnosis of PDCA [44-47], CA125 is mostly applied in ovarian cancer [48]. Many studies have proved the prognostic value of CA19-9 and CA125 in PDCA [44-47]. It was reported that CA125 levels specifically reflect the metastasis associated burden of pancreatic cancer in patients with advanced disease, as well as the presence of occult metastasis in patients with clinically localized tumors [46]. Our data indicate that both CA125 (*P* = 0.001) and CA19-9 (*P* = 0.023) are independent predictive indicator for LN metastasis, which is consisted with Zhou's study [47]. Therefore, CA125 and CA19-9 may be promising, noninvasive, LN metastasis associated biomarker for PDCA.

This study presents several limitations. Firstly, our study is hospital based on a single institution, not a population-based study. Such design might have introduced a selection bias because of differential referral patterns. However, such limitation was outweighed by its strength. We do not believe that we introduced an ascertaining bias for misdiagnosis, because all patients had both pathologically and clinically confirmed LN metastasis and PDCA. Secondly, the lost of information on some quantifiable factors, as well as the small size of patients, precluded us from estimating the magnitude of PDCA risk for LN metastasis associated with these factors. Although our study has a relatively small sample size, the incidence of LN metastasis is high in PDCA after operation, and given that this is a major focus of this study, the sample size is probably adequate.

In conclusion, the present study showed that NLR, CA125 and CA19-9 could be convenient, reliable and economical predictive tools to distinguish PDCA patients with LN metastasis between those without LN metastasis in the study, which are useful for further planning of selective neoadjuvant therapy or nodal dissection before surgery.

## MATERIALS AND METHODS

### Ethical statement

The study was approved by the Clinical Ethics Committee of Peking University Third Hospital. The patients’ data were analyzed anonymously because written consent was not obtained from all participants.

### Study population and design

All participants were enrolled from Peking University Third Hospital (Beijing, China) from February 2005 to December 2014. The patients who underwent a curative resection of primary PDCA and whose diagnoses were confirmed by pathological examination were involved in this study. Preoperative cytological examination was not regularly performed. Patients whose pre-operative examination showed celiac artery or superior mesenteric artery tumor invasion or distant metastasis (liver, lung and bone metastases) were not performed curative operation, no matter them have regional lymph node metastasis or not. However, four patients were found have liver metastasis during operation but not pre-operative examination. No other situation such as occult peritoneal disease which would explain high CA 125 during the included cases, as all of them was carefully checked. Eight PDAC patients who underwent neoadjuvant chemotherapy or chemoradiation therapy were excluded from the study. The NLR and PLR of the 159 PDAC patients were calculated in the database. All surgical specimens were evaluated pathologically to determine the extent of tumor differentiation, LN metastases, and surgical margins following surgery. The pathological stage of PDAC was determined according to the American Joint Committee on Cancer (AJCC)7th Edition [49]. According to the previously studies, the preoperative NLR was calculated as the neutrophil count divided by lymphocyte count [50], and the preoperative PLR was calculated as the platelet count divided by the lymphocyte count [51].

For the first 3 years following surgery, the patients were followed up at intervals of at most 3 months. These follow up visits consisted of a physical examination, laboratory examination including the measurement of tumor markers, and computed tomography (CT) or magnetic resonance imaging (MRI). In some cases, ultrasound or positron emission tomography-computed tomography (PET-CT) was also used. From the 3-year time point following surgery, the patients with no sign of metastasis or recurrence were monitored at 3- to 6-month intervals. The patients were followed up until death or December 31, 2015. The metastasis time was measured from the day of surgery to the date of the diagnosis of metastasis. The overall survival time was measured from the day of surgery to the date of death or the last follow-up.

### Statistical analysis

The Mann-Whitney U-test and a box-plot were used to describe the normality of each continuous parameter's distribution. Quantitative results are reported in the form of the means ± standard deviation. The ROC curve was used to estimate the performance of NLR and PLR. χ^2^-test was used for univariate analysis of LN metastasis. The associations between clinical and histopathological parameters with OS and metastasis were analyzed using Kaplan-Meier curves and compared by the log-rank test. Univariate and multivariate Cox-regression analyses were performed to determine the effects of possible prognostic factors on metastasis after curative surgery. Hazard ratios (HRs) estimated from the Cox analysis were shown as relative risks with corresponding 95% confidence intervals (CIs). The associations between the clinical and histopathological parameters with LN metastasis were evaluated by both univariate analysis and multivariate logistic regression analysis. On the basis of the univariate analysis, those variables with P value less than 0.05 were included in the multivariate logistic regression analysis to confirm independent variables. The forward stepwise method was utilized to eliminate variables that did not show significant information. Hazard ratios (HRs) and 95% confidence intervals (CIs) of each independent variable were calculated routinely. All analyses were carried out using the SPSS 22.0 statistical software (SPSS, IL, USA). *P* < 0.05 was considered statistically significant.

### Institutional review board statement

This study was approved by the Clinical Ethics Committee of Peking University Third Hospital.
